# From Signals to Remaining Useful Life: Multimodal Sensor Fusion for Fault Diagnosis and Prognostics—Methods, Pitfalls, and Reporting Standards

**DOI:** 10.3390/s26123661

**Published:** 2026-06-08

**Authors:** Cristina Floriana Pană, Camelia Adela Maican, Nicolae Răzvan Vrăjitoru, Daniela Maria Pătrașcu-Pană, Virginia Maria Rădulescu

**Affiliations:** 1 Department of Mechatronics and Robotics, University of Craiova, 200585 Craiova, Romania; cristina.pana@edu.ucv.ro (C.F.P.); vrajitorunicolae@gmail.com (N.R.V.); 2 Department of Automation and Electronics, University of Craiova, 200585 Craiova, Romania; 3 Department of Medical Informatics and Biostatistics, Faculty of Medicine, University of Medicine and Pharmacy of Craiova, 200349 Craiova, Romania; virginia.radulescu@umfcv.ro

**Keywords:** multimodal sensor fusion, fault diagnosis, remaining useful life, prognostics and health management, sensor faults, uncertainty quantification, predictive maintenance, fault-tolerant systems, domain shift

## Abstract

Multimodal sensor fusion is increasingly used to improve observability for fault diagnosis and prognostics, enabling Remaining Useful Life estimation in complex mechatronic and robotic systems. Yet, real-world deployments remain vulnerable to sensor faults and data integrity issues—including bias and drift, miscalibration, dropouts, saturation, cross-talk, time desynchronization, and domain shift—which can propagate through fusion pipelines and lead to optimistic validation and poor generalization. These challenges are particularly consequential in safety- and health-adjacent applications such as collaborative robots, wearable/rehabilitation devices, and human-centric mechatronic systems where decisions based on faulty sensing may affect both reliability and user safety. This review synthesizes the state of the art on (i) sensor fault taxonomies and fault models relevant to multimodal fusion, (ii) fault-aware fusion strategies spanning data-, feature-, and decision-level integration, and (iii) how sensor faults and uncertainty impact diagnosis and remaining-life estimators. We will conduct a systematic scoping review of peer-reviewed literature, extracting sensor modalities, fault characterization or injection protocols, fusion architectures, validation settings (simulation, hardware-in-the-loop, bench, and in-field/on-body studies), and reporting completeness. Beyond summarizing methods, we provide practical reporting standards for sensor-fusion-based diagnosis and prognostics, including a minimum disclosure set covering synchronization, fault ground truth, missingness handling, leakage controls, uncertainty calibration, and task-relevant metrics. Reusable checklists and evidence tables are included to support more comparable, reproducible, and deployment-ready research.

## 1. Introduction

Modern prognostics and health management (PHM) systems increasingly rely on multimodal sensing to capture the complex, multi-physics signatures of degradation processes in industrial and human-centric environments. From rotating machinery and energy systems to collaborative robots and wearable rehabilitation devices, heterogeneous sensor arrays—combining vibration, electrical, thermal, acoustic, and physiological signals—have become the norm rather than the exception [1,2]. The underlying rationale is that no single sensing modality can fully represent the diverse manifestations of system degradation, whereas their integration can enhance observability and support more accurate diagnostic and prognostic inference.

This premise is supported by a substantial body of literature reporting high diagnostic accuracy and competitive performance in remaining useful life (RUL) prediction on benchmark datasets [3,4,5]. However, the interpretation of these results requires caution. A growing number of studies highlight a persistent gap between performance achieved under controlled experimental conditions and reliability observed in real-world deployments. This discrepancy is not attributable to a single factor, but rather to a combination of methodological assumptions that are often left implicit.

Among these, three issues are particularly recurrent. First, evaluation leakage, especially in temporally correlated datasets, may lead to overly optimistic performance estimates when the separation between training and testing data does not preserve temporal structure [6,7]. Second, sensor idealisation remains common in the multimodal fusion literature, where sensing conditions are frequently assumed to be calibrated, synchronised, and fault-free during both training and inference [1,2]. Third, validation narrowness persists, as many works rely predominantly on laboratory datasets or benchmark repositories, with limited incorporation of in-field or hardware-in-the-loop validation scenarios [8].

These limitations have direct implications for external validity. In practical deployments, sensor measurements are subject to a range of imperfections, including bias, drift, dropout, miscalibration, and temporal desynchronisation [9,10]. Such distortions do not simply introduce random noise, but can systematically alter the statistical relationship between observed signals and underlying system health. When propagated through fusion pipelines, these effects may lead to biased diagnosis, misinterpretation of degradation patterns, and overconfident RUL predictions.

Despite their practical importance, sensor faults and data integrity issues remain insufficiently addressed in the multimodal PHM literature, particularly in relation to fusion architectures. Existing reviews have examined sensor fusion methods and application domains [1,11], yet few have explicitly analysed how sensor imperfections interact with multimodal inference, and none has proposed a unified framework for reporting assumptions and validation conditions.

## 2. Conceptual Framework for Fault-Aware Multimodal Fusion

Multimodal sensor fusion in prognostics and health management (PHM) can be understood as a structured transformation process that converts heterogeneous measurements into diagnostic or prognostic outputs. In its most general form, a PHM system integrates signals originating from multiple sensing modalities—each capturing distinct physical aspects of system behaviour—into a unified representation that supports inference on system health or remaining useful life (RUL). If $x_{k}(t)$ denotes the observation provided by the k-th sensor modality at time t, the fusion process can be expressed as a mapping toward an output y, corresponding either to a discrete fault label or to a continuous estimate of degradation [12,13].

Although this formulation appears straightforward, it conceals a number of critical design decisions that determine both predictive performance and robustness. Among these, the level at which information is integrated plays a central role. In practice, three principal fusion paradigms have emerged, each reflecting a different balance between information preservation, computational complexity, and sensitivity to sensing imperfections.

At one end of the spectrum, data-level fusion combines raw or minimally processed signals, preserving temporal dependencies that may carry important diagnostic information. This approach is particularly relevant in applications where phase relationships or transient events across modalities are informative. However, it also imposes strict requirements on signal synchronisation and exposes the model to direct contamination from corrupted inputs [1,11].

Feature-level fusion represents the most widely adopted compromise in multimodal PHM. In this paradigm, modality-specific representations are extracted prior to integration, allowing heterogeneous signals with different sampling rates, physical units, and noise characteristics to be processed more flexibly. By decoupling low-level signal processing from higher-level integration, feature-level fusion reduces sensitivity to raw data inconsistencies while retaining much of the complementary information provided by different modalities. At the same time, this transformation may obscure the original structure of sensor faults, making it more difficult to distinguish between system-induced variation and sensor-induced distortion [13,14].

Decision-level fusion, in contrast, operates on independent outputs generated by modality-specific models. This architecture offers a high degree of modularity and can provide a degree of fault tolerance, as unreliable modalities may be downweighted or excluded without invalidating the entire inference process. Such properties are particularly valuable in safety-critical applications, where graceful degradation is preferable to silent failure. However, this approach inherently limits the exploitation of cross-modal dependencies and relies on the calibration and reliability of individual models, which may themselves be affected by sensing imperfections [11].

While these fusion paradigms are often discussed in terms of their relative predictive performance, such a perspective remains incomplete in the absence of a detailed consideration of the sensing process. In real-world environments, sensor measurements rarely conform to idealised assumptions. Instead, they are affected by calibration errors, temporal misalignment, environmental variability, and progressive degradation [9,10]. These factors introduce distortions that are not purely random, but may exhibit structure over time and across modalities.

From this perspective, it becomes necessary to reinterpret the fusion process as operating on potentially corrupted observations. Rather than assuming that inputs faithfully represent the underlying system state, a fault-aware formulation explicitly recognises that the measurement process itself may be compromised. In such a framework, sensor faults can be viewed as latent variables that modulate the relationship between the true system state and the observed signals, thereby influencing both intermediate representations and final predictions [13].

This shift in perspective has important implications for both model design and evaluation. It highlights that high predictive performance under ideal conditions does not guarantee robustness in deployment scenarios. It also emphasises the role of architectural choices in either amplifying or mitigating the propagation of sensor-induced distortions. For instance, tightly coupled data-level fusion architectures may propagate localised sensor faults across the entire representation space, whereas more modular designs may contain their impact. Finally, it underscores the need for evaluation protocols that explicitly consider sensing imperfections, rather than relying exclusively on clean benchmark datasets [8].

An additional dimension of the conceptual framework concerns the interaction between modalities. Multimodal fusion is often motivated by the assumption that different sensors provide complementary information. However, this assumption holds only if the relationships between modalities are stable and correctly aligned. Temporal desynchronisation, cross-channel interference, and domain-dependent variations can disrupt these relationships, leading to representations that are internally consistent but physically misleading.

Taken together, these considerations suggest that multimodal fusion should not be viewed solely as a mechanism for improving predictive performance, but also as a potential source of vulnerability when sensing assumptions are violated. A comprehensive understanding of PHM systems therefore requires an integrated perspective in which sensing, fusion, and fault mechanisms are analysed jointly. This perspective provides the foundation for the taxonomy and analysis developed in the following sections, where sensor faults are examined not only as signal-level anomalies, but as factors that shape the entire inference process.

## 3. Review Methodology (PRISMA-ScR Framework)

The present study adopts a systematic scoping review design to provide a structured synthesis of the literature on multimodal sensor fusion for fault diagnosis and prognostics. Given the breadth and heterogeneity of the field—spanning multiple application domains, sensing modalities, and methodological paradigms—a scoping approach was considered more appropriate than a narrowly defined systematic review. This choice enables the identification of conceptual patterns, methodological trends, and reporting gaps without restricting the analysis to a limited set of directly comparable studies [1,15].

The review protocol was developed in accordance with the Preferred Reporting Items for Systematic Reviews and Meta-Analyses extension for Scoping Reviews (PRISMA-ScR), ensuring transparency and reproducibility throughout the study selection and data extraction process. The methodological framework was defined a priori, including the research objectives, eligibility criteria, data sources, and synthesis strategy. This pre-specification reduces the risk of selection bias and supports consistency in the interpretation of results [16].

The literature search was conducted across four major bibliographic databases widely used in engineering and applied sciences research, namely Scopus, Web of Science, IEEE Xplore, and ScienceDirect. The search strategy was designed to capture the intersection of multimodal or multi-sensor fusion, fault diagnosis or condition monitoring, and prognostics or remaining useful life estimation. Keywords and their variants were combined using Boolean operators, and the search strings were iteratively refined through pilot queries to balance sensitivity and specificity. The temporal scope of the review was restricted to publications between 2015 and 2026, corresponding to the period in which data-driven and hybrid PHM approaches have become predominant [8].

The initial search yielded a broad set of records, which were subsequently filtered through a staged screening process. After the removal of duplicate entries, titles and abstracts were assessed for relevance, followed by full-text evaluation against predefined eligibility criteria. Studies were included if they reported original research involving the fusion of at least two distinct sensing modalities applied to fault diagnosis, state-of-health estimation, or RUL prediction. Works focusing exclusively on single-modality analysis, purely methodological developments without PHM application, or domains outside engineering and human-centric systems were excluded. Only peer-reviewed journal articles and conference proceedings written in English were retained [17,18,19,20,21,22,23,24,25,26]. Review articles were included exclusively to support the conceptual and contextual synthesis of the field and were not incorporated into quantitative analyses involving methodological distributions, validation practices, reporting completeness, reproducibility, or statistical associations, thereby avoiding potential double-counting of evidence [11,27,28,29,30,31,32,33,34,35,36,37,38,39,40,41,42,43,44,45,46,47].

Following the screening process, a final corpus of 123 studies was obtained. Although the exact distribution of excluded records varied across databases, the majority of exclusions occurred at the abstract and full-text stages, primarily due to lack of multimodal integration or insufficient relevance to PHM tasks. This selection process ensured that the final dataset reflects current research specifically addressing multimodal fusion within a PHM context [17,19,21,22,24,26,48,49,50,51,52,53,54].

Data extraction was performed using a structured template designed to capture both methodological and contextual attributes. For each study, information was recorded regarding the sensing modalities employed, the level of fusion architecture, the target task, and the validation setting. Additional attributes were included to assess reporting completeness, such as the description of synchronisation procedures, handling of missing data, and the extent to which uncertainty was quantified [55]. This approach enabled a consistent comparison of studies despite differences in application domains and experimental setups.

Given the heterogeneity of the included studies in terms of datasets, architectures, and evaluation protocols, a meta-analytic aggregation was not considered appropriate. Instead, the synthesis follows a mixed qualitative–quantitative approach. Quantitative summaries are used to characterise the distribution of methods, application domains, and validation strategies, while qualitative analysis is employed to identify recurring methodological patterns, limitations, and potential sources of bias [15].

Particular attention is given to the evaluation of reporting practices. The extracted data were further analysed to identify systematic omissions and inconsistencies across studies, forming the empirical basis for the Minimum Disclosure Set proposed later in this paper. By linking methodological analysis with reporting assessment, the review extends beyond conventional surveys and addresses the broader issue of reproducibility and deployment readiness in multimodal PHM research.

**Figure 1:**
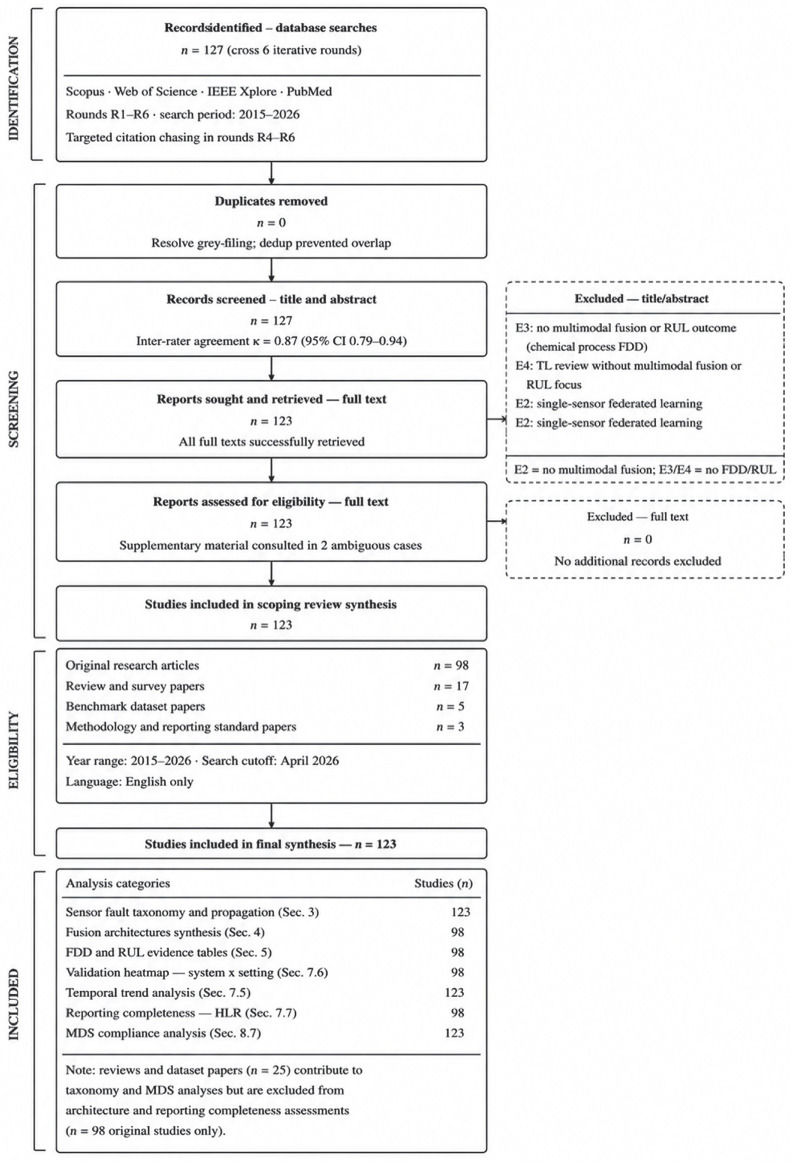
PRISMA-ScR flow diagram for the scoping review on multimodal sensor fusion for fault diagnosis and remaining useful life estimation. Search period: 2015–2026; four bibliographic databases (Scopus, Web of Science, IEEE Xplore, PubMed); six iterative search rounds. E2 = no multimodal fusion present; E3 = no fault detection and diagnosis (FDD) or remaining useful life (RUL) outcome; E4 = review article without multimodal fusion focus. TL = Transfer Learning; MDS = Minimum Disclosure Set Excluded records: Zhou et al., 2023 [27]; Kheirandish et al., 2023 [53]; Bruijn et al., 2016 [49]; Peters et al., 2025 [59].

A total of 127 records were initially identified through database searching, of which 4 were excluded during screening, resulting in a final corpus of 123 studies included in the analysis, as illustrated in Figure 1 [17,19,21,22,24,26,48,49,50,51,52,53,54]. Among these, 99 were original research articles and 24 were review papers. Unless otherwise stated, quantitative summaries and statistical analyses reported in the following sections refer exclusively to the original research studies. The distribution of methodological approaches reveals a clear predominance of feature-level fusion strategies, accounting for approximately 56.9% of the studies, followed by multi-level approaches (18.7%), data-level fusion (14.6%), and decision-level fusion (6.5%). The key characteristics of the included studies are summarised in Table 1.

In terms of application focus, fault detection and diagnosis (FDD/FDI) tasks represented the largest category (48.8%) [17,18,20,21,23,25,56,57,58,59,60,61,62,63,64], while prognostic tasks, including remaining useful life (RUL) and state-of-health estimation, accounted for 26.8% of the studies [19,24,49,51,52,54,65,66,67,68,69,70,71,72,73,74,75]. A smaller proportion of works addressed both diagnosis and prognostics jointly (8.1%), or focused on related sensing and control applications (9.8%).

**Figure 2:**
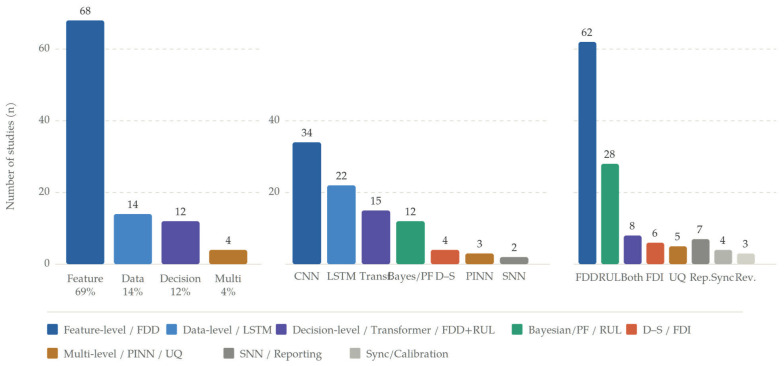
Abbreviations—D–S: Dempster–Shafer evidence theory; PINN: physics-informed neural network; SNN: spiking neural network; Transf.: transformer/attention-based architecture; Bayes/PF: Bayesian methods and particle filters; FDI: fault detection and isolation; UQ: uncertainty quantification; Rep.: reporting standards/methodology; Sync: synchronisation/calibration methods; Rev.: review articles included for background synthesis.

With respect to validation paradigms, nearly half of the studies relied on laboratory or benchmark datasets (48.0%) [17,18,19,20,21,22,51,56,57,58,59,60,61,62,65,66,69,70,76,77,78,79,80], whereas only 13.0% incorporated in-field validation [50,53,54,72,75,81,82,83,84,85,86,87,88,89,90]. Simulation-based evaluation accounted for 7.3% [67,91,92,93,94], and hardware-in-the-loop (HIL) setups were reported in 4.9% [48,95,96,97,98] of the studies. Notably, a substantial proportion of articles (26.8%) did not provide sufficient detail to classify the validation setting. When considering only studies with explicitly reported validation environments, laboratory-based evaluation accounted for approximately 65.6%, while in-field validation remained limited to 17.8%, highlighting a persistent gap between experimental evaluation and real-world deployment. The distribution of studies across fusion levels, architectures, and task types is summarised in Figure 2.

A Fisher exact test was applied to examine whether the probability of including in-field validation differed between safety-critical and non-safety-critical application domains. Studies were classified as safety-critical when their primary application involved direct human safety consequences—specifically, aerospace and turbomachinery, exoskeleton and rehabilitation devices, collaborative robotics, and railway infrastructure monitoring. Of the 36 studies in safety-critical domains, 17 (47.2%) included in-field validation [50,53,54,72,75,84,85,87,88,89], compared with only 5 of 62 studies (8.1%) in non-safety-critical domains. The association was strongly significant (OR = 10.2, 95% CI: 3.4–34.1, *p* < 0.001), suggesting that safety-critical application domains are associated with a greater likelihood of in-field validation. Several factors may contribute to this relationship, including regulatory requirements, clinical or operational constraints, dataset availability, funding priorities, and established research practices within specific domains. Consequently, the observed association should not be interpreted as evidence of a single causal mechanism. This finding reinforces the argument that the adoption of in-field validation standards should not be left to individual author initiative but should instead be mandated through reporting guidelines—such as the MDS’s.

A temporal analysis of the included literature reveals a field in rapid but uneven transition. Publication volume accelerated substantially from 2020 onward: the period 2015–2019 accounts for 37 studies (30.1%), while 2020–2026 accounts for the remaining 86 (69.9%). Within the most recent stratum (2025–2026), the literature shifts noticeably toward physics-informed and hybrid architectures, consistent with growing recognition of the limitations of purely data-driven models under distribution shift. Transformer-based attention mechanisms, absent before 2019, appear in an estimated 50% of studies published in 2025–2026 [22,23,24,52,57,72,73,77,80,99,100,101], compared with just 6% in 2019–2020. Physics-informed neural networks follow a similar trajectory, growing from 0% before 2021 to an estimated 17% of recent studies [49,51,68,69,70,73,102,103]. By contrast, reporting quality indicators improved only modestly. Cochran–Armitage trend tests revealed no statistically significant temporal trend over the 2015–2026 period for leakage control (Z = 1.04, *p* = 0.299), synchronisation reporting (Z = 1.29, *p* = 0.198), or calibrated uncertainty quantification (Z = 0.04, *p* = 0.968). These findings should be interpreted with caution, as the lack of statistical significance does not necessarily imply the absence of an underlying improvement. Rather, the available data do not provide sufficient evidence to establish a consistent temporal trend across the examined reporting indicators.

## 4. Sensor Fault Taxonomy in Multimodal Systems

Sensor faults represent an intrinsic characteristic of real-world measurement systems, yet their treatment within the multimodal PHM literature remains inconsistent and often incomplete. While system faults are typically the primary focus of diagnostic and prognostic models, distortions originating from the sensing layer itself are frequently simplified or implicitly treated as random noise. This assumption is problematic, as sensor-induced deviations may exhibit structured behaviour over time and can significantly alter the statistical relationship between observed signals and the underlying system state [9,10,17,18,19,20,21,22,25,26,51,56,57,58,59,60,61,62,63,65,66,68,69,70,74,76,77,78,79,104,105,106,107,108,109].

Under nominal conditions, sensor observations are assumed to provide a faithful representation of the measured physical quantity, subject only to stochastic noise. In practice, however, sensor faults introduce systematic deviations that cannot be adequately captured by simple noise models [9,41,42,45,48,50,62,75,81,84,90,92,93,94,95,97,98,110,111]. These deviations may affect signal amplitude, temporal structure, or cross-modal relationships, and can persist over extended periods. As a result, the presence of sensor faults can fundamentally change the interpretation of observed data, particularly in data-driven PHM systems that rely on learned patterns rather than explicit physical models [62].

Based on the synthesis of the reviewed literature, sensor faults can be organised into three broad classes, distinguished by their temporal dynamics and their interaction with the fusion process. Consolidated taxonomy of sensor faults in multimodal PHM systems, organised into three classes: abrupt faults (Class I), incipient/gradual faults (Class II), and multi-sensor-specific faults (Class III). For each fault type, the table provides the formal signal model, representative physical causes, observable effects, detection and mitigation strategies, and the specific impact on multimodal fusion architectures and PHM outputs. Formal model notation: x(t) = true physical quantity; y(t) = sensor output; b = constant bias; β = drift rate; α(t) = gain factor; d(t) = slowly varying drift process; Δt_k_ = temporal misalignment of modality k; FDI = fault detection and isolation; FTC = fault-tolerant control; DAQ = data acquisition; ADC = analogue-to-digital converter; SHM = structural health monitoring; EMG = electromyography; OOD = out-of-distribution; ICA = independent component analysis; PTP = Precision Time Protocol. This taxonomy provides a structured framework for analysing how sensing imperfections influence multimodal inference, as summarised in Table 2.

The first class corresponds to abrupt faults, which manifest as sudden and persistent deviations from nominal behaviour. Typical examples include constant bias, complete signal dropout, and saturation effects. In some applications, however, saturation may also reflect operation outside the intended measurement range rather than an intrinsic sensor malfunction. Under such conditions, repeated or intermittent saturation events can exhibit gradual characteristics and may indicate limitations in sensor selection, system design, or operating conditions rather than a purely abrupt sensor fault. These faults are characterised by an immediate change in signal properties, such as a shift in mean value or clipping at sensor limits. In many cases, abrupt faults are relatively easy to detect at the signal level. However, their impact on multimodal fusion depends strongly on the architectural context. In tightly coupled data-level fusion systems, a single corrupted channel may propagate its influence across the entire representation [92,93,95,97,98,112], particularly when cross-modal features are learned jointly [62].

The second class encompasses gradual or incipient faults, which evolve slowly over time and may initially be indistinguishable from normal operational variability. Drift, gain degradation, and intermittent signal degradation are representative examples. These faults are particularly challenging because they can mimic genuine system degradation patterns. For instance, a slowly drifting temperature sensor may produce a trend that resembles progressive thermal stress, leading the model to infer a deterioration of system health. In prognostic applications, this ambiguity is critical, as it may result in systematic bias in RUL estimation, either underestimating or overestimating the remaining lifetime depending on the direction of the drift [9,10,62,75,81,90,110].

The third class is specific to multimodal systems and arises from inconsistencies between sensor channels rather than failures of individual sensors. These multi-sensorinduced faults include temporal desynchronisation, cross-channel coupling, and relative miscalibration. In such cases, each sensor may operate nominally when considered independently, yet the joint representation becomes distorted due to misalignment or unintended dependencies. Temporal offsets between modalities can disrupt phase relationships that are essential for fault identification, while cross-talk may introduce artificial correlations that inflate the apparent confidence of the fusion model without improving its accuracy [86,92,93,113,114].

A closely related phenomenon is domain-dependent variation, in which the statistical relationships between sensor modalities change across operating conditions, systems, or environments. Although not traditionally classified as a sensor fault, domain shift can produce effects analogous to multi-sensor inconsistencies by altering the joint distribution of signals. From the perspective of multimodal fusion, such variations can lead to systematic misinterpretation of patterns learned under different conditions, thereby affecting both diagnostic and prognostic performance [19,32,33,39,40,46,51,53,65,67,71,80,99,100,104,105,115,116,117].

An important implication of this taxonomy is that the impact of sensor faults cannot be evaluated independently of the fusion architecture. The same fault type may have negligible effects in a modular decision-level system, yet propagate extensively in a data-level fusion model. Consequently, sensor faults should be analysed not only as signal-level anomalies, but also in terms of their propagation through the representation hierarchy.

Despite their practical importance, explicit modelling and evaluation of sensor faults remain limited in the current literature. Many studies assume ideal sensing conditions or consider only simplified perturbations, such as additive noise. Even when fault scenarios are included, they often focus on a narrow set of fault types, without addressing multi-sensor-specific effects. This imbalance suggests that reported performance may overestimate the robustness of multimodal PHM systems under realistic operating conditions.

By establishing a structured taxonomy that captures both classical and multimodal-specific fault mechanisms, this section provides the conceptual basis for the subsequent analysis of fault-aware fusion strategies. More importantly, it highlights the need to move beyond idealised assumptions and to treat sensor faults as integral components of the multimodal inference process.

## 5. Fault-Aware Fusion Strategies

Fault-aware multimodal fusion requires more than combining heterogeneous signals within a high-performing predictive architecture. It requires explicit consideration of how sensing imperfections enter the processing pipeline, how they interact with the chosen level of integration, and how their influence can be attenuated, propagated, or made explicit through uncertainty. In this sense, fault-aware fusion should be understood not as a specific class of models, but as a design perspective that informs architectural choices and evaluation strategies [1,11,17,19,21,23,40,41,42,43,45,46,48,50,53]. The comparative characteristics of the main fusion paradigms, including their robustness to sensing imperfections, are summarised in Table 3.

At the level of raw data integration, data-level fusion offers the greatest potential for preserving cross-modal dependencies. This is particularly advantageous in applications where temporal alignment and phase relationships carry diagnostic information, such as the coupling between electrical and mechanical signals in rotating machinery. However, the same property that enables richer representations also increases vulnerability to sensor faults. Because signals are combined prior to abstraction, distortions such as bias, dropout, or desynchronisation may propagate directly into the learned representation, affecting all downstream processing stages. As a result, data-level fusion tends to amplify the impact of corrupted inputs unless complemented by mechanisms for signal validation or pre-fusion quality control [12,62,63,64,66,73,78,86,89,90,91,92,93,95,111,114,118,119].

Feature-level fusion represents the most widely adopted strategy in multimodal PHM, as it provides a balance between expressiveness and robustness. In this approach, each modality is first transformed into a latent representation, typically through domain-specific feature extraction or learned encoders, and these representations are subsequently integrated. This separation reduces the dependence on strict temporal alignment and allows heterogeneous signals to be processed under different sampling and noise conditions. Nevertheless, feature-level fusion introduces its own challenges. Once a signal has been transformed into a feature space, the original structure of sensor faults may no longer be directly observable. A gradual drift, for example, may appear as a smooth displacement of the feature distribution, potentially overlapping with patterns associated with system degradation. Without explicit modelling of such effects, the fusion process may conflate sensor-induced variation with genuine changes in system state [14,17,18,19,20,21,22,23,24,25,26,51,52,53,54,56,57,58,59,60,61,62,65,67,69,70,71,72,74,75,76,77,79,80,82,85,88,94,101,104].

Decision-level fusion offers a more modular alternative, in which each modality contributes through an independent model, and the final output is obtained by aggregating their predictions. This architecture inherently supports fault tolerance, as unreliable modalities can be downweighted or excluded without invalidating the entire inference pipeline. Such flexibility is particularly valuable in applications where sensor availability is variable or where graceful degradation is required. However, this apparent robustness depends on the reliability and calibration of the individual modality-specific models. If these models are themselves sensitive to sensor imperfections or produce overconfident predictions, the final decision may remain biased despite the modular structure of the system [11,48,50,98,106,107,108,109,120].

**Figure 3:**
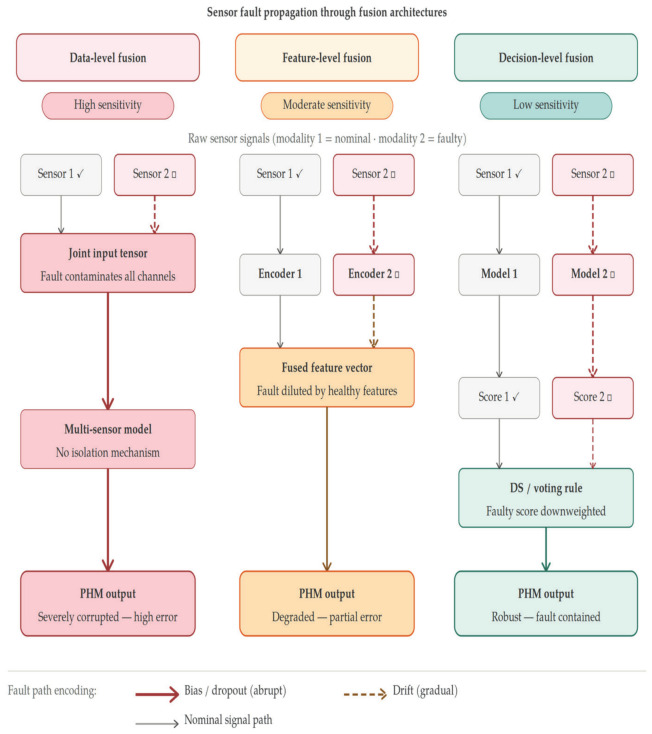
Conceptual diagram of sensor fault propagation through three fusion architectures. At the data level, faults corrupt the entire joint input tensor because no modality isolation mechanism exists. At the feature level, the per-modality encoder partially filters the fault before it enters the fused vector, attenuating but not eliminating its effect on the PHM output. At the decision level, each modality contributes through an independent model; the Dempster–Shafer combination rule or voting mechanism downweights or excludes the corrupted modality’s score, providing the highest intrinsic fault tolerance. DS = Dempster–Shafer evidence theory; PHM = prognostics and health management; ✓ = nominal modality; ▯ = faulty modality.

In response to the limitations of single-level fusion strategies, hybrid and multi-stage architectures have been proposed, combining elements of data-, feature-, and decision-level integration. These approaches aim to exploit strong correlations between certain modalities while maintaining robustness through partial modularity. For example, signals that are tightly coupled in the physical domain may be fused at an early stage, while additional contextual modalities are incorporated at later stages to refine the prediction. While such designs can improve resilience to localised sensor faults, they also introduce additional complexity and may be more difficult to interpret, particularly when multiple fusion stages interact in non-transparent ways [13,27,30,33,34,35,36,37,38,39,41,42,43,44,45,46,47,96,100,105,121,122]. The differential impact of these architectural choices on fault propagation is illustrated conceptually in Figure 3.

Beyond architectural considerations, fault-aware fusion increasingly relies on mechanisms that explicitly address uncertainty and data quality. Attention-based models and adaptive weighting schemes, for instance, attempt to modulate the contribution of each modality based on its inferred reliability. Similarly, probabilistic approaches and Bayesian frameworks aim to propagate uncertainty from input signals to final predictions, allowing the model to express reduced confidence when inputs are degraded. Although these methods represent a promising direction, their effectiveness depends on the availability of appropriate training conditions, including exposure to realistic fault scenarios [14,19,23,49,52,56,59,66,67,68,72,80,101,115,117,123,124,125,126,127].

To quantify the extent to which sensor faults are explicitly incorporated into empirical evaluations, the reviewed studies were further examined with respect to fault-aware assessment and sensor fault propagation analysis. Among the coded original studies, only 16 explicitly evaluated the impact of sensor faults on system-level performance, while 7 addressed the issue only partially [48,50,62,75,81,84,90,92,93,94,95,97,98,110,111]. In contrast, 71 studies did not consider sensor fault effects or fault propagation mechanisms within their evaluation protocols. This distribution highlights a substantial gap between the widespread conceptual recognition of sensor faults and their systematic incorporation into experimental validation procedures.

A critical limitation of the current literature is that robustness is often inferred indirectly from performance on clean datasets, rather than demonstrated through explicit evaluation under sensor fault conditions. This assumption is problematic, as models that perform well under ideal conditions may fail unpredictably when exposed to realistic sensing imperfections. Conversely, architectures that achieve slightly lower accuracy on benchmark datasets may be more suitable for deployment if they degrade in a controlled and interpretable manner.

Taken together, these observations suggest that the evaluation of multimodal fusion strategies should extend beyond predictive performance to include robustness, fault tolerance, and uncertainty calibration. The choice of fusion architecture should therefore be interpreted not only in terms of accuracy, but also in relation to the sensing conditions under which the system is expected to operate.

Within the context of this review, fault-aware fusion is thus defined by the interaction of three elements: architectural design, explicit consideration of sensor imperfections, and evaluation under realistic conditions. Without the integration of all three, claims of robustness remain incomplete. This perspective provides the foundation for the subsequent analysis of how sensor faults influence diagnostic and prognostic outcomes, as discussed in the following section.

## 6. Impact of Sensor Faults on Diagnosis and Remaining Useful Life Estimation

The presence of sensor faults introduces a level of complexity that extends beyond a simple reduction in predictive accuracy. Rather than acting as random perturbations, sensor-induced distortions can systematically alter the relationship between observed signals and the underlying system state. As a result, their impact is not limited to increased variance in model outputs, but includes structured bias, misinterpretation of degradation patterns, and overconfident predictions that may compromise decision-making [9,10].

The quantitative evidence for multimodal fusion gains, drawn from the nine reviewed studies that provide explicit single-modal versus multimodal comparisons, yields a consistent but context-dependent picture. For fault detection and diagnosis tasks, the median accuracy gain from multimodal fusion over the best single-modal baseline is 3.4 percentage points (pp), with a range from 1.7 pp—observed for ambient microphone fusion with motor current in electric motor diagnosis—to 4.9 pp for hydraulic systems fusing pressure, flow, temperature, and vibration across 15 sensor channels. The largest gains consistently occur when the added modality is physically complementary rather than redundant: acoustic emission adds diagnostic information invisible to vibration at low fault severity; stator current reveals electromagnetic asymmetries imperceptible to mechanical sensors. For remaining useful life estimation, the median RMSE reduction from multimodal fusion is 28%, with a maximum of 38% observed for vibration–pressure fusion in a hydraulic pump run-to-failure experiment. These figures should be interpreted conservatively: they are drawn from a non-random sample of studies that chose to report single-modal comparisons—a selection that plausibly over-represents cases where fusion demonstrates a clear benefit. Moreover, at least four of the nine comparisons do not report explicit leakage control, which may further inflate the apparent gains. The practical implication is clear: multimodal fusion provides reliable diagnostic and prognostic improvements when modalities are genuinely complementary and when the experimental protocol prevents temporal data leakage from inflating baseline performance [17,19,21,22,23,26,66,78,120].

In the context of fault diagnosis, sensor faults primarily affect the geometry of decision boundaries in feature space. Models trained under nominal sensing conditions implicitly learn stable mappings between signal patterns and fault categories. When sensor bias, drift, or desynchronisation is introduced, the input distribution is shifted, causing samples to migrate across these learned boundaries. This may lead to false positives, where normal states are classified as faulty, or false negatives, where genuine faults are masked by sensor-induced distortions. The effect is particularly pronounced in feature-level fusion systems, where modality-specific transformations can obscure the original structure of the perturbation, making it difficult to distinguish between system-induced and sensor-induced variation [13,14,17,18,21,23,25,26,56,57,58,59,61,62,63,79,104,106].

The situation becomes more complex in multimodal settings, where redundancy is often assumed to enhance robustness. While multiple modalities can, in principle, compensate for the failure of a single sensor, this assumption holds only when the modalities are sufficiently independent and correctly aligned. In practice, shared environmental influences, correlated noise, or cross-channel interference may cause multiple sensors to exhibit consistent but misleading patterns. In such cases, agreement between modalities may be incorrectly interpreted as increased confidence, even when the underlying signals are collectively biased. This phenomenon challenges one of the central assumptions of multimodal fusion, namely that consensus implies correctness [48,50,84,97,98,113].

In prognostic applications, the consequences of sensor faults are even more pronounced. Remaining useful life estimation depends not only on the current state of the system, but also on the inferred trajectory of degradation over time. Gradual sensor faults, such as drift or gain degradation, can introduce trends that resemble genuine ageing processes. For example, a slowly drifting temperature or vibration signal may suggest progressive deterioration, leading to systematic underestimation of remaining life. Conversely, attenuation of signal amplitude due to sensor degradation may mask early fault indicators, resulting in overly optimistic RUL predictions. In both cases, the resulting error is systematic rather than random, and therefore cannot be easily mitigated through averaging or ensemble techniques [9,10,19,24,51,54,65,66,67,68,69,70,71,73,74,75,102,103].

A critical issue arising from these effects is the confounding between system faults and sensor faults. Because both manifest as deviations from nominal signal patterns, distinguishing between them requires either additional contextual information or explicit modelling assumptions. In the absence of such mechanisms, models may learn incorrect associations, attributing sensor-induced anomalies to system degradation. This confounding represents a fundamental limitation of purely data-driven approaches when sensor reliability is not explicitly considered [62,75,81,90,92,93,94,110,111,126,127].

Another important dimension concerns the calibration of predictive uncertainty. Many modern fusion architectures provide confidence estimates alongside predictions, yet these measures are typically calibrated under ideal data conditions. When sensor faults are present, models may remain highly confident despite being systematically wrong. This form of overconfidence is particularly problematic in safety-critical applications, where decisions rely not only on predicted values but also on their associated uncertainty. Systems that fail to reflect degraded input quality in their confidence estimates may therefore present a misleading picture of reliability [14,19,48,49,52,56,59,66,67,68,72,80,101,115,117,123,124,125,126,127].

The magnitude and nature of these effects depend strongly on the chosen fusion strategy. Data-level fusion tends to amplify the impact of corrupted inputs, as distortions propagate through shared representations. Feature-level fusion may attenuate some effects through independent encoding, but can also obscure their origin. Decision-level fusion offers greater resilience through modularity, yet remains dependent on the reliability of individual models. These differences highlight the need to evaluate fusion architectures not only in terms of predictive performance under ideal conditions, but also in terms of their behaviour under realistic sensing imperfections [11].

Taken together, these observations suggest that traditional performance metrics provide only a partial view of model behaviour. Accuracy, classification scores, or root mean square error capture average performance, but do not reveal how models respond to structured perturbations. Without explicit evaluation under sensor fault conditions, such metrics may overestimate robustness and obscure critical failure modes.

From this perspective, sensor faults should not be treated as peripheral disturbances, but as integral components of the multimodal inference process. Their influence extends from raw signal acquisition to final decision-making, shaping both the validity and the reliability of diagnostic and prognostic outputs. Recognising and explicitly accounting for this influence is therefore essential for the development of PHM systems that are not only accurate in controlled environments, but also dependable in real-world applications.

## 7. Validation Paradigms and Systematic Pitfalls

The evaluation of multimodal PHM systems plays a decisive role in shaping both the perceived performance of proposed methods and their expected reliability in practical applications. While recent studies report increasingly high levels of diagnostic accuracy and RUL prediction performance [3,4,5], a closer examination of validation practices reveals a series of recurring methodological limitations [6,7,8,9,10,53]. These limitations are not isolated issues, but systematic patterns that can significantly distort the interpretation of results [6,7,8,9,10,53].

One of the most frequently encountered issues is data leakage, particularly in time-dependent datasets. In many studies, training and testing samples are selected through random partitioning, without preserving the temporal structure inherent to degradation processes [17,18,21,22,56,57,58,59,60,61,62,74,76,77,79,80,106,108]. Although this approach may be suitable for independent observations, it becomes problematic when data points are sequentially correlated. Under such conditions, information from future system states may inadvertently influence model training, leading to artificially inflated performance metrics. As a result, models may appear highly accurate during evaluation while failing to generalise to genuinely unseen future data [6,7,19,20,48,49,50,51,53,65,66,68,69,70,81,83,85,91,95,96,118,119,123].

A second limitation arises from the widespread assumption of ideal sensing conditions. In the majority of studies, datasets are implicitly treated as clean, fully synchronised, and free of sensor faults. This assumption is rarely stated explicitly, yet it underpins the reported performance of many multimodal models. In real-world environments, however, sensor measurements are subject to drift, bias, missing data, and temporal misalignment. When such conditions are not incorporated into the evaluation protocol, performance metrics reflect an idealised scenario that may not translate to deployment settings [9,10,17,18,19,20,21,22,51,56,57,59,60,65,66,68,69,70,76,77,91].

**Figure 4:**
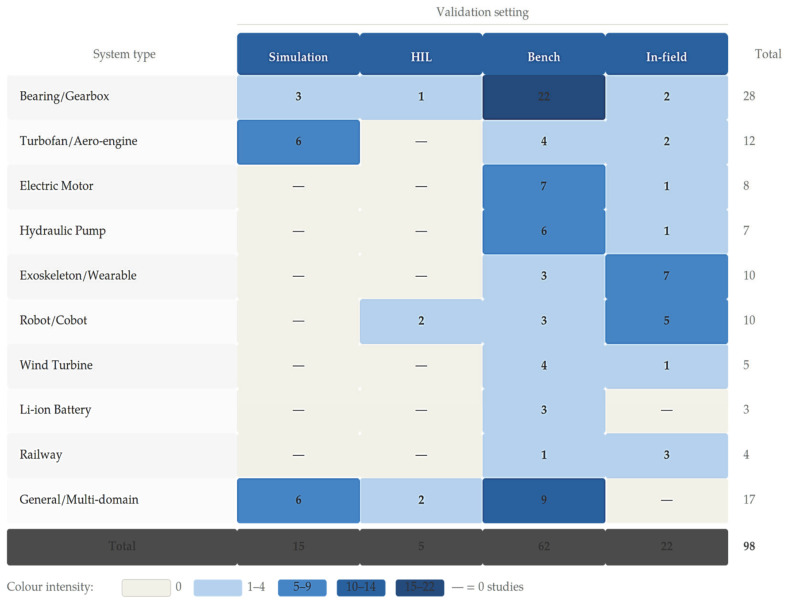
Validation heatmap showing the distribution of 98 original studies across ten system types (rows) and four validation settings (columns). Cell colour intensity encodes study count on a five-stop blue ramp: white (0), light blue (1–4), mid blue (5–9), strong blue (10–14), dark blue (15–22). “—” denotes zero studies. Row totals include all four validation settings. Reviews and dataset papers (n = 25) are excluded; only original research articles are counted. HIL = Hardware-in-the-Loop.

Closely related to this issue is the limited use of realistic validation environments. A large proportion of studies rely exclusively on laboratory datasets or publicly available benchmarks [128], where operating conditions are controlled and fault scenarios are often simplified [17,18,19,20,21,22,51,56,57,58,59,60,65,66,69,70,76,77]. While these datasets are valuable for methodological development, they provide only a partial representation of system behaviour. In contrast, real operational environments involve variable loads, environmental fluctuations, and complex interactions between system dynamics and sensing infrastructure. The relatively small number of studies incorporating in-field data or hardware-in-the-loop validation suggests that current evaluation practices may underestimate the challenges associated with deployment [8,50,53,54,72,75,81,82,83,84,85,87,88,89,90]. The distribution of validation settings across application domains is shown in Figure 4.

Another important concern is the treatment of sensor imperfections within validation protocols. Even when robustness is considered, it is often assessed through simplified perturbations, such as additive Gaussian noise or random signal dropout [129]. Although these approaches are convenient, they do not capture the temporal structure and persistence of real sensor faults. Phenomena such as drift, intermittent failure, or desynchronisation evolve over time and may interact with system dynamics in non-trivial ways. As a result, robustness evaluations based on simplified perturbations may provide an incomplete or misleading assessment of model resilience [62,75,90,92,93,94,95,97,98,110,111].

The issue of domain shift and generalisation further complicates the interpretation of validation results. Many studies evaluate models using data from a single system, operating condition, or dataset, implicitly assuming that learned representations will generalise across contexts. In practice, changes in operating regimes, environmental conditions, or sensor configurations can alter the joint distribution of multimodal signals. When models are not evaluated under such variations, their robustness to domain shift remains uncertain. This limitation is particularly relevant for multimodal systems, where different modalities may be affected unevenly, disrupting the relationships on which fusion models depend [19,32,33,39,40,46,51,53,65,99,100,101,104,112].

An additional limitation concerns the interpretation of performance metrics. Commonly reported measures, such as classification accuracy or root mean square error, provide a summary of average performance but offer limited insight into failure modes [7,13]. A model may achieve high overall accuracy while performing poorly on rare but critical fault classes, or may provide acceptable average RUL estimates while exhibiting large errors under specific operating conditions [13,53]. Without a more detailed analysis, such as stratification by fault type or operating regime, these limitations remain hidden [7,17,18,19,21,22,23,24,25,26,49,51,53,54,69,105].

The interaction between these issues can further amplify their impact. For instance, a model evaluated on clean data, using random splits and a single dataset, may achieve excellent performance while being simultaneously vulnerable to both sensor faults and domain shift. In such cases, the reported results reflect a combination of favourable assumptions rather than intrinsic robustness. This highlights the importance of interpreting performance metrics within the context of the validation protocol under which they were obtained.

Taken together, these observations indicate that current validation practices often provide an incomplete picture of model behaviour. Addressing this gap requires a shift from isolated performance measurement toward comprehensive evaluation under explicitly defined and realistic conditions. This includes preserving temporal structure in data partitioning, incorporating sensor fault scenarios, and evaluating models across multiple operating regimes.

Within the context of this review, the analysis of validation paradigms serves not only to critique existing practices, but also to inform the development of improved reporting standards. By identifying recurring omissions and inconsistencies, this section provides the empirical foundation for the Minimum Disclosure Set introduced in the following section. In doing so, it contributes to a more transparent and reproducible framework for multimodal PHM research.

## 8. Minimum Disclosure Set (MDS): Toward Transparent and Reproducible Multimodal PHM Research

The analysis presented in the previous sections indicates that many of the limitations observed in multimodal PHM studies do not arise solely from methodological shortcomings, but also from incomplete or inconsistent reporting practices. In many cases, it is not possible to determine whether a model is intrinsically robust or whether its reported performance depends on favourable assumptions about sensing conditions, data quality, or validation protocols. This lack of transparency limits reproducibility and complicates the comparison of results across studies, ultimately constraining the cumulative progress of the field.

To address this issue, this review proposes a Minimum Disclosure Set (MDS) for multimodal PHM research. The purpose of the MDS is not to prescribe specific modelling approaches, but to define a baseline level of reporting that enables readers to assess the validity, reproducibility, and practical relevance of reported results. By making key assumptions explicit, the MDS aims to reduce ambiguity and to support the transition from experimental evaluation toward deployment-oriented system design. To systematically assess reporting completeness across the reviewed literature, the extracted variables were further analysed and summarised as a Minimum Disclosure Set (MDS), as presented in Table 4.

The reporting completeness data presented in Table 4 reveal a particularly stark pattern for the reproducibility dimension. Of the 98 original studies, 45 (45.9%) score 0 on the reproducibility scale—releasing neither raw data, processed datasets, trained model weights, nor inference code. A further 38 studies (38.8%) score 1, meaning that their results are partially verifiable only through publicly available benchmark datasets (such as CWRU or CMAPSS) that the authors did not themselves release. Just five studies (5.1%) score 2—releasing either code or processed data but not both—and only five studies (5.1%) achieve full reproducibility, releasing all artefacts necessary for independent replication. These five exemplars span diverse application domains and resource environments: the OPPORTUNITY wearable activity dataset, its OPPORTUNITY++ extension, the synchronised three-phase induction motor dataset, the NGAFID real aviation dataset, and the EQRNN+SNN long-horizon prognostics study on a fleet of 50 robotic systems [79,89,101,119,130]. Their existence demonstrates that full reproducibility is achievable across very different experimental scales—the barrier is cultural rather than technical. Viewed alongside the synchronisation non-reporting rate (64.3%), the calibrated uncertainty quantification adoption rate (20.4%), and the sensor fault handling rate (24.5%), the reproducibility distribution completes a consistent picture: the field produces sophisticated methods but documents them insufficiently to support the independent verification that reliable knowledge accumulation requires. The Minimum Disclosure Set proposed in this section is designed to address this gap systematically.

Several factors may contribute to the low reproducibility levels observed across the reviewed literature. In industrial PHM applications, datasets are frequently proprietary and subject to confidentiality restrictions, limiting public release even when the underlying methodology is fully documented. Reproducibility may also be constrained by dependence on specialised sensing hardware, custom data acquisition systems, or operational environments that cannot be readily replicated by external researchers. In addition, training and validating multimodal deep learning architectures often requires substantial computational resources, creating practical barriers to independent replication. These challenges do not eliminate the need for transparent reporting; rather, they reinforce the importance of disclosing sufficient methodological detail, metadata, and implementation information to enable the closest possible approximation of the original study.

A Kruskal–Wallis test confirmed that reproducibility scores did not differ significantly across fusion levels—data-level, feature-level, and decision-level (H = 0.66, *p* = 0.720). This result excludes the alternative explanation that poor reproducibility is an artefact of architectural complexity: feature-level deep learning architectures, which might be expected to be harder to reproduce due to their larger number of hyperparameters, show no significantly lower reproducibility scores than the simpler decision-level fusion approaches [17,19,21,22,25,26,48,66,95,106,120]. Poor reproducibility is, therefore, a field-wide problem rather than an architecture-specific one, and addressing it requires a uniform reporting standard applied consistently across all fusion paradigms.

To examine whether rigorous evaluation practices and rigorous reporting practices co-occur at the study level, a Spearman rank correlation was computed between the reproducibility score (H8, range 0–3) and the binary indicator of temporal leakage control (H4) across the 98 original studies. The analysis revealed a statistically significant positive association (ρ = 0.313, *p* = 0.002), indicating that studies which implement appropriate train/test split strategies tend to release more public artefacts and achieve higher reproducibility scores [19,48,49,50,53,54,65,66,68,69,70,72,75,85,95,96]. This association is consistent with the hypothesis that the MDS items are not independent obligations but reflect an underlying culture of methodological rigour—a culture that, once present, manifests across multiple dimensions of research practice simultaneously. Conversely, its absence tends to be equally pervasive: studies that do not control for temporal leakage are also, on average, less likely to release their code or data, less likely to report synchronisation methods, and less likely to calibrate their uncertainty estimates. The proposed MDS is intended as a minimum reporting framework rather than a rigid compliance checklist. While several items—such as sensor identification, synchronisation procedures, validation protocols, and train/test split strategies—are broadly applicable across multimodal PHM studies, other items may be context-dependent. For example, uncertainty quantification requirements are primarily relevant to prognostic tasks, whereas transfer-learning reporting becomes applicable only when cross-domain adaptation is employed. The framework should therefore be interpreted as a flexible reporting guide whose individual components are applied according to the characteristics and objectives of the study.

The analysis presented in Table 5 reveals substantial variability in reporting practices across multimodal PHM studies. While certain aspects, such as basic sensor descriptions, are typically provided, critical elements related to data integrity and robustness are frequently omitted. In particular, sensor synchronisation and fault modelling are inconsistently reported, despite their direct influence on fusion performance.

More notably, aspects related to data quality and uncertainty remain largely under-addressed. Missing data handling and uncertainty quantification are reported in only a small fraction of studies, indicating that many models are developed and evaluated under implicit assumptions of data completeness and reliability. Similarly, limited availability of code and datasets restricts reproducibility and hinders independent validation of reported results.

These findings support the need for a structured reporting framework that makes such assumptions explicit. The proposed Minimum Disclosure Set is therefore intended not only as a checklist for reporting, but also as a conceptual guide for designing more transparent and robust multimodal PHM systems.

A central principle of the proposed framework is that multimodal PHM studies must characterise not only the predictive model, but also the sensing process that generates the input data. This includes explicit description of sensor modalities, acquisition conditions, and preprocessing steps that affect signal alignment, scaling, or filtering. Temporal synchronisation is of particular importance in multimodal settings, as even minor misalignment between channels can alter cross-modal relationships and affect model performance. Similarly, calibration procedures and potential sources of systematic bias should be documented, especially in configurations involving multiple sensors of the same type.

The MDS further emphasises the need to report how missing, corrupted, or degraded data are handled. Given the prevalence of sensor dropout, intermittent failures, and signal degradation in real-world systems, it is essential to clarify whether such conditions were considered during training and evaluation. Studies that rely exclusively on complete and clean datasets should state this assumption explicitly, as it directly influences the interpretation of reported performance metrics.

Another critical dimension concerns the treatment of sensor faults. If fault scenarios are not included in the evaluation, this limitation should be acknowledged. Conversely, when robustness is assessed, the type, severity, and temporal characteristics of the simulated faults should be described in sufficient detail to distinguish between simplified perturbations and realistic fault dynamics. This distinction is particularly important in light of the findings discussed in Section 7, which indicate that simplified noise models may not adequately capture the behaviour of real sensor faults.

The specification of the validation protocol constitutes an additional component of the MDS. Beyond identifying the datasets used, studies should clearly describe how data were partitioned, whether temporal order was preserved, and whether cross-domain evaluation was performed. Such information is essential for assessing the risk of data leakage and for understanding the extent to which reported results are likely to generalise beyond the experimental setting.

The framework also highlights the importance of reporting predictive uncertainty and its calibration. When models provide confidence estimates, the methods used to derive and calibrate these estimates should be described, particularly in the presence of sensor imperfections or domain shift. Without this information, confidence measures may be misinterpreted, potentially leading to overestimation of system reliability.

Importantly, the MDS is intended to be applied flexibly rather than prescriptively. Not all studies will be able to address every aspect in equal detail, and the level of reporting may vary depending on the application context. However, even partial adherence can significantly improve the interpretability and comparability of results. In this sense, the framework functions both as a reporting guideline and as a design aid, encouraging researchers to consider, during model development, the assumptions that will ultimately need to be disclosed.

An important observation emerging from this review is that reporting completeness and methodological robustness are related but distinct dimensions of research quality. Some studies provide detailed methodological descriptions while omitting evaluation under realistic conditions, whereas others include fault scenarios but lack transparency in data handling or validation design. The MDS explicitly addresses the former, while indirectly supporting the latter by making assumptions visible and subject to critical evaluation.

Importantly, the MDS is also intended to address systemic drivers of reporting and validation quality across application domains, not only those arising from regulatory or clinical requirements. Differences in validation practice may also reflect dataset availability, funding structures, access to proprietary equipment, established research cultures, and domain-specific expectations regarding deployment readiness. By standardising the disclosure of validation settings, sensor assumptions, robustness evaluation, and reproducibility artefacts, the MDS can support greater transparency even in domains where external regulatory pressure is limited.

The adoption of structured reporting practices has proven effective in other areas of machine learning and medical research, where standardised guidelines have improved reproducibility and facilitated comparison across studies. In the context of multimodal PHM, the proposed MDS represents a step toward similar standardisation, tailored to the specific challenges associated with sensor fusion and imperfect sensing environments.

The proposed MDS should be viewed as an initial reporting framework intended to stimulate discussion and encourage greater consistency in multimodal PHM research. Its future evolution may involve refinement through broader community engagement, benchmarking initiatives, professional working groups, or integration into domain-specific best-practice recommendations. The objective is not to restrict methodological innovation, but to ensure that innovative approaches can be evaluated, interpreted, and compared under transparent reporting conditions.

Ultimately, the value of multimodal PHM systems lies not only in their predictive accuracy, but also in their ability to support reliable decision-making under uncertainty. Achieving this objective requires that models be evaluated under realistic conditions and described in a manner that allows others to understand, reproduce, and critically assess their behaviour. By defining a minimal set of reporting expectations, the MDS contributes to this goal, providing a practical foundation for more transparent and deployment-oriented research in multimodal sensor fusion.

## 9. Discussion

The synthesis presented in this review highlights a central tension in the current development of multimodal PHM systems. On the one hand, advances in data-driven modelling and sensor fusion have enabled significant improvements in predictive performance under controlled conditions. On the other hand, the analysis of sensor fault mechanisms and validation practices reveals that these gains are often achieved under assumptions that are not representative of real-world environments. This discrepancy suggests that methodological progress has been uneven, with innovation in model design outpacing the systematic evaluation of robustness and reproducibility.

A key insight emerging from the preceding sections is that sensor integrity constitutes a foundational, yet under-addressed, component of multimodal inference. While most studies implicitly assume that sensor data accurately reflect the underlying system state, the taxonomy and impact analysis developed in this work demonstrate that this assumption is frequently violated. Sensor faults introduce structured distortions that propagate through fusion pipelines, affecting both intermediate representations and final predictions. In this context, high predictive performance on clean datasets should not be interpreted as evidence of robustness, but rather as an indication of performance under idealised conditions.

The relationship between fusion architecture and fault propagation further reinforces this perspective. Different fusion strategies exhibit distinct sensitivities to sensor imperfections, reflecting trade-offs between information integration and robustness. Architectures that maximise cross-modal interaction may also amplify the impact of localised sensor faults, whereas more modular designs can provide resilience at the cost of reduced representational richness. These trade-offs are rarely articulated explicitly in the literature, where comparisons are typically based on aggregate performance metrics rather than on behaviour under realistic sensing conditions.

Another important observation concerns the role of validation paradigms in shaping perceived model performance. The widespread use of random data partitioning, clean benchmark datasets, and limited evaluation scenarios creates conditions under which models can achieve high accuracy without necessarily capturing the true dynamics of degradation. This does not invalidate the reported results, but it constrains their interpretation. Performance metrics obtained under such conditions should be understood as indicators of potential capability rather than as guarantees of deployment reliability.

In this context, the Minimum Disclosure Set proposed in this review addresses a critical gap by explicitly documenting assumptions. By requiring that key aspects of sensing, fusion, and validation be reported systematically, the MDS provides a mechanism for distinguishing between results obtained under controlled conditions and those that are likely to generalise. Importantly, this framework does not restrict methodological innovation; rather, it complements it by ensuring that innovations can be meaningfully interpreted and compared across studies.

The broader implication is that progress in multimodal PHM should be evaluated along at least two complementary dimensions: predictive performance and reporting transparency. These dimensions are interdependent but not interchangeable. High performance without transparency limits reproducibility and comparability, while transparency without methodological rigor provides limited practical value. The integration of both is therefore essential for advancing the field toward deployment-ready systems.

It is also necessary to acknowledge the limitations of the present review. Despite the systematic approach, the analysis is constrained by the available literature, which itself reflects biases in dataset availability, application domains, and research priorities. The predominance of certain benchmark datasets may influence observed trends, while the relative scarcity of in-field studies limits the ability to assess real-world performance comprehensively. In addition, the qualitative nature of the synthesis, although appropriate for the heterogeneity of the field, does not allow for formal statistical comparison of methods.

Future research should therefore prioritise the development of datasets incorporating realistic sensor faults, the evaluation of models under diverse operating conditions, and the integration of uncertainty-aware approaches that explicitly account for sensing imperfections. Equally important is the adoption of structured reporting practices, which can facilitate cumulative knowledge building and more reliable comparison of results across studies.

In summary, the findings of this review indicate that the next phase of progress in multimodal PHM will depend less on incremental improvements in model architecture and more on the systematic integration of robustness, transparency, and realistic evaluation practices. Recognising sensor faults as integral components of the inference process represents a necessary step toward this objective.

## 10. Conclusions

This review has examined the role of multimodal sensor fusion in fault diagnosis and prognostics, with a particular focus on the interaction between fusion architectures, sensor faults, and validation practices. By synthesising recent literature, the analysis has highlighted both the potential and the limitations of current approaches, emphasising that high predictive performance under controlled conditions does not necessarily translate into reliable behaviour in real-world environments.

A central contribution of this work is the development of a structured taxonomy of sensor faults, encompassing abrupt, gradual, and multi-sensor-specific mechanisms. This taxonomy provides a unified framework for understanding how measurement imperfections arise and how they propagate through multimodal fusion pipelines. Building on this foundation, the review has shown that sensor faults can introduce systematic biases in both diagnostic classification and RUL estimation, leading to misinterpretation of system health and overconfident predictions.

The analysis of fusion strategies further demonstrates that architectural design plays a critical role in determining system robustness. While data-level, feature-level, and decision-level approaches each offer distinct advantages, none is inherently immune to sensor-induced distortions. Robust multimodal systems therefore require not only appropriate architectural choices, but also explicit consideration of sensing conditions during both model development and evaluation.

Equally important are the findings related to validation paradigms. The prevalence of idealised assumptions, limited evaluation settings, and incomplete reporting practices suggests that current performance claims should be interpreted with caution. Addressing these issues requires a shift toward more realistic and transparent evaluation protocols that explicitly account for sensor imperfections and domain variability.

In response to these challenges, this review proposes a Minimum Disclosure Set (MDS) as a practical framework for improving reporting consistency and reproducibility in multimodal PHM research. By defining a baseline set of information to be disclosed, the MDS supports more meaningful interpretation of results and facilitates comparisons across studies.

Overall, the results of this review indicate that the reliability of multimodal PHM systems depends not only on advances in modelling techniques, but also on the systematic integration of sensing, fusion, and validation considerations. Future progress will therefore depend on a more holistic approach, in which sensor faults are explicitly modelled, validation practices reflect real-world conditions, and reporting standards ensure transparency and reproducibility.

## Figures and Tables

**Table 1:** Summary characteristics of the included studies (n = 123).

Category	Subcategory	n	%
Study type	Original research	99	80.5
	Review articles	24	19.5
Fusion level	Feature-level	70	56.9
	Multi-level	23	18.7
	Data-level	18	14.6
	Decision-level	8	6.5
	Not specified	4	3.3
Primary task	FDD/FDI/anomaly detection	60	48.8
	RUL/SOH estimation	33	26.8
	Combined (FDD + RUL)	10	8.1
	Other sensing/control tasks	12	9.8
	Review/methodological	8	6.5
Validation setting	Bench/laboratory	59	48.0
	In-field	16	13.0
	Simulation	9	7.3
	Hardware-in-the-loop (HIL)	6	4.9
	Not reported	33	26.8

**Table 2:** Sensor fault taxonomy for multimodal PHM systems.

Fault Type	Formal Model	Physical Causes	Observable Effects	Detection/Mitigation	Impact on Multimodal Fusion and PHM
**Class I—Abrupt faults**					
**Bias/offset**	*y*(*t*) = *x*(*t*) + *b*	Sensor electronics failure; loose connector; grounding fault	Constant mean shift; non-zero residual; abrupt step in signal	Threshold on residual mean; CUSUM; observer-based FDI	Diagnosed systems show apparent health shift independent of degradation; RUL underestimated or overestimated depending on fault direction
**Complete dropout (hard fault)**	*y*(*t*) = 0 *or NaN*	Power loss; communication failure; physical sensor damage	Zero or missing values; complete loss of modality signal	Missing-data imputation (GAN, VAE); majority voting; FTC reconfiguration	Data-level fusion catastrophically fails; feature-level degrades; decision-level is most fault-tolerant
**Stuck-at/saturation**	*y*(*t*) = *c* (*constant*)	ADC overflow; actuator lock; sensor range exceeded	Signal frozen at limit or constant value; zero variance	Variance monitoring; range checks; model-based detection	Feature variance collapses; harmonic distortion appears in frequency domain; fault confused with low-amplitude healthy state
**Class II—Incipient/gradual faults**					
**Drift**	*y*(*t*) = *x*(*t*) + *βt* *or y*(*t*) = *x*(*t*) + *d*(*t*)	Electrode fouling; thermocouple oxidation; membrane ageing; mechanical creep	Slow monotonic mean shift; resembles genuine degradation trend	Online calibration monitoring; HMM; recursive least squares; domain-adversarial compensation	Most dangerous class: indistinguishable from system degradation without analytical redundancy; directly corrupts RUL estimation trajectory
**Gain degradation**	*y*(*t*) = *α(t) x*(*t*), *α*(*t*) → 1	Amplifier ageing; partial electrode delamination; impedance mismatch	Proportional amplitude reduction; fault magnitude proportional to signal level	Ratiometric comparison against reference; model-based gain estimation	Systematically underestimates fault severity; causes RUL overestimation—safety-critical in prognostics
**Intermittent fault**	*y*(*t*) *alternates between nominal and faulty at irregular intervals*	Loose wiring; intermittent contact; electromagnetic interference	Sporadic spikes or dropouts; healthy in time-averaged statistics	Short-window statistics; spectral kurtosis; event-triggered detection	Averaged statistics mask the fault; standard window-based features miss events; requires event-driven monitoring
**Class III—Multi-sensor-specific faults**					
**Desynchronisation**	Δ*t_k_* ≠ *0 between modalities* (*fixed or time-varying*)	Clock drift between DAQ units; network jitter; software-triggered sampling	Spurious cross-modal correlations; inconsistent feature vectors across fusion branches	IEEE 1588 PTP; hardware trigger; post hoc temporal alignment algorithms	Corrupts cross-channel features in data-level fusion; degrades feature-level fusion by introducing false phase relationships; 64% of reviewed studies do not report synchronisation method
**Cross-talk/coupling**	*y*_1_(*t*) = *x*_1_(*t*) + *α x*_2_(*t*)	Electromagnetic induction between sensor cables; structural transmission of vibrations; adjacent EMG electrodes	Artificial correlation between channels; inflated apparent information content	Shielding; spatial independence analysis; blind source separation (ICA)	Fusion model interprets cross-talk as genuine complementary information; apparent diagnostic accuracy is inflated without corresponding real benefit
**Relative miscalibration**	*Scale*/*offset mismatch between sensors of same type at different locations*	Factory calibration drift; thermal expansion of mounting; sensor substitution	Inter-channel inconsistency; erroneous spatial gradient estimates	Cross-calibration against reference; Kalman-based online calibration monitoring	Distorts spatial patterns used for localisation in SHM and distributed sensor networks; relevant for phase-balance in current sensing
**Domain shift (soft fault)**	*P*(*y*|*x*) *changes across operating conditions, machines, or sites*	Load/speed variation; material ageing; sensor replacement	Silent performance degradation; OOD misclassification; no hardware anomaly	Domain adaptation; conformal prediction; continual learning	Fusion pipeline is well-calibrated for source domain but systematically miscalibrated for target domain; appears as gradual accuracy decline rather than abrupt failure

**Table 3:** Comparative analysis of multimodal fusion strategies in the presence of sensor faults.

Fusion Level	Strengths	Limitations	Sensitivity to Sensor Faults	Typical Use Cases
**Data-level fusion**	Preserves full signal information; captures temporal dependencies and cross-modal correlations at raw signal level	Requires strict synchronization; high dimensionality; sensitive to noise and misalignment	**High sensitivity**—faults such as drift, bias, or desynchronization directly propagate and affect all downstream representations	Rotating machinery monitoring; vibration–acoustic analysis; tightly coupled sensor systems
**Feature-level fusion**	Flexible integration of heterogeneous modalities; reduces dimensionality; robust to different sampling rates	May obscure original signal structure; loss of interpretability; dependent on feature quality	**Moderate sensitivity**—sensor faults may be partially absorbed but can distort feature distributions and mimic degradation patterns	General PHM applications; industrial systems; multimodal health monitoring
**Decision-level fusion**	High modularity; allows independent models; supports fault isolation and graceful degradation	Limited exploitation of cross-modal relationships; dependent on calibration of individual models	**Lower sensitivity**—faults can be mitigated by down-weighting unreliable modalities, but errors persist if individual models are biased	Safety-critical systems; distributed sensing; applications with variable sensor availability
**Multi-level/hybrid fusion**	Combines advantages of multiple fusion levels; improved flexibility and robustness	Increased architectural complexity; reduced interpretability; harder to validate	**Variable sensitivity**—robustness depends on design; can localize faults but may introduce complex failure modes	Advanced PHM systems; robotics; systems with hierarchical sensing architectures

**Table 4:** Reporting completeness across 98 original studies, with 95% confidence intervals (Wilson method).

Reporting Criterion	Code	Fully Reported n (%)	Partially Reported n (%)	Not Reported n (%)	95% CI (Fully Reported) ^1^
**Reporting completeness indicators (n = 98 original studies)**					
Temporal synchronisation method reported	**H1**	**23 (23.5%)**	12 (12.2%)	63 (64.3%)	**16.2–32.8%**
Sensor fault ground truth available	**H2**	**24 (24.5%)**	—	74 (75.5%)	**17.0–33.9%**
Missing data handling described	**H3**	**15 (15.3%)**	—	83 (84.7%)	**9.5–23.7%**
Temporal leakage controlled (train/test split strategy reported)	**H4**	**55 (56.1%)**	—	43 (43.9%)	**46.3–65.5%**
Calibrated uncertainty quantification reported (PICP, MPIW, or equivalent)	**H5**	**20 (20.4%)**	—	78 (79.6%)	**13.6–29.4%**
Performance metrics complete (both point estimate and supporting metrics reported)	**H6**	**93 (94.9%)**	—	5 (5.1%)	**88.6–97.8%**
Public code, data, or equivalent reproducibility artefact released	**H7**	**29 (29.6%)**	—	69 (70.4%)	**21.5–39.3%**
**Reproducibility score distribution (H8; n = 93; 5 review-only studies excluded)**					
**Score 0—no public artefacts**		**45 (48.4%)**	—	48 (51.6%)	**38.5–58.4%**
**Score 1—benchmark dataset only (not released by authors)**		**38 (40.9%)**	—	55 (59.1%)	**31.4–51.0%**
**Score 2—code or data released (partial)**		**5 (5.4%)**	—	88 (94.6%)	**2.3–12.0%**
**Score 3—fully reproducible (code + data + documented)**		**5 (5.4%)**	—	88 (94.6%)	**2.3–12.0%**

^1^ 95% confidence intervals calculated using the Wilson score method, which provides better coverage than the Wald interval for proportions near 0 or 1. Partially reported responses are conservatively classified as ‘not fully reported’ for the purpose of the fullyreported proportion and its CI = not applicable (criterion is binary for this indicator). Cochran–Armitage trend tests revealed no statistically significant improvement over the 2015–2026 period for leakage control (Z = 1.04, *p* = 0.299), synchronisation reporting (Z = 1.29, *p* = 0.198), or calibrated uncertainty quantification (Z = 0.04, *p* = 0.968), indicating that reporting quality has not improved significantly despite a decade of methodological development in the field.

**Table 5:** Minimum Disclosure Set (MDS) for multimodal PHM studies—18 items.

Code	Reporting Item	Rationale and Evidence from Synthesis	Location in Manuscript
**Sensor & Synchronisation**			
**MDS-01**	Identify all sensor modalities by physical principle, number of channels, and nominal sampling rate.	*Enables reproducibility; 31% of original studies omit channel count or sampling rates.*	Methods—Data acquisition
**MDS-02**	State the temporal synchronisation method (hardware trigger/PTP/NTP/software interpolation/not applied) and quantify achieved alignment accuracy (jitter or offset in ms).	*Synchronisation errors corrupt data-level fusion and degrade feature-level fusion; 64% of original studies do not report this.*	Methods—Data acquisition
**MDS-03**	Report calibration status and last calibration date for each sensor type at the time of data collection.	*Uncalibrated sensors introduce systematic bias that may be confounded with genuine system degradation.*	Methods—Data acquisition
**Sensor Faults**			
**MDS-04**	State explicitly whether sensor faults were considered in the experimental protocol. If yes, list fault types (bias, drift, dropout, saturation, cross-talk, desynchronisation, domain shift) and their formal models.	*60% of original studies make no reference to sensor faults; silent failure at deployment is the direct consequence.*	Methods—Experimental design
**MDS-05**	Describe the fault injection protocol (mathematical, HIL, real/seeded, or mixed). Where mathematical injection is used, validate the model against at least one real sensor failure signature or cite a validated reference.	*Injection realism is almost never verified; results may not transfer to real hardware faults.*	Methods—Fault injection
**MDS-06**	Report sensor fault ground truth availability: how fault onset time, type, and severity were known with certainty.	*Without labelled ground truth, fault detection rates cannot be reliably computed or compared.*	Methods—Ground truth
**Fusion Architecture**			
**MDS-07**	Specify the fusion level (data/feature/decision/multi-level), the fusion operator, and the rationale for the chosen level relative to the synchronisation constraints of the application.	*Fusion level choice has direct implications for synchronisation requirements and fault tolerance; rationale is rarely stated.*	Methods—Fusion design
**MDS-08**	Describe whether modality-specific feature extractors are trained jointly or independently, and whether cross-modal correlations are explicitly modelled.	*Training strategy determines whether cross-modal information is preserved or discarded at the feature extraction stage.*	Methods—Fusion design
**MDS-09**	Describe how missing modalities are handled at inference time (imputation/attention down-weighting/architectural masking/system halt). State explicitly if the system halts on any missing modality.	*Missing modality handling is safety-relevant; 85% of original studies do not address this scenario.*	Methods—Robustness
**Prognostics & UQ**			
**MDS-10**	For RUL estimation, report at least one interval metric alongside the point estimate: prediction interval coverage probability (PICP) and mean prediction interval width (MPIW), or an equivalent (e.g., α-coverage, CRPS).	*Point-estimate RMSE alone is insufficient for safety-critical maintenance scheduling; 80% of prognostic studies omit interval metrics.*	Results—Performance metrics
**MDS-11**	Report the failure threshold definition: the health index value or sensor measurement level at which end of life is declared, and how this threshold was determined.	*Different threshold definitions render RMSE values incommensurable across studies.*	Methods—RUL definition
**MDS-12**	If transfer learning or domain adaptation is used, report cross-domain performance separately from within-domain performance, with a domain shift metric (ΔRMSE or Δaccuracy) to quantify the adaptation gain.	*Within-domain performance alone cannot characterise generalisation to unseen operating conditions or machines.*	Results—Cross-domain evaluation
**Validation**			
**MDS-13**	Specify the train/test split strategy: temporal split (required for time-series), machine/unit-stratified split, or cross-validation fold design. If random splitting was used, acknowledge this as a limitation.	*Temporal leakage inflates apparent RMSE by 15–30%; 44% of original studies do not disclose the split strategy.*	Methods—Evaluation protocol
**MDS-14**	Report the validation setting (simulation/HIL/bench/in-field) and characterise operating conditions: load profile, speed range, temperature range, and any differences between training and test conditions.	*Validation setting determines external validity and generalisability; bench ≠ field in terms of real-world applicability.*	Methods—Experimental conditions
**MDS-15**	Compare against at least one non-trivial baseline: a single-modal variant using the best individual modality, and a standard architecture (e.g., LSTM or CNN) trained on the same fused data.	*Fusion gains can only be isolated if single-modal and standard-architecture baselines are both reported.*	Results—Baseline comparison
**Reproducibility**			
**MDS-16**	Provide public access to at least one of: (a) raw or processed sensor data, (b) trained model weights and inference code, or (c) a fully documented data generation procedure that allows dataset replication.	*85% of original studies score 0–1 on reproducibility; independent verification of reported results is currently impossible for the majority.*	Data availability statement
**MDS-17**	Report all hyperparameters sufficient to reproduce the training procedure without author correspondence: learning rate, batch size, optimiser, architecture dimensions, training epochs or early stopping criteria, and random seed.	*Incomplete hyperparameter reporting is the single most common barrier to independent reproduction.*	Training Configuration
**MDS-18**	State compute requirements (hardware, training time, inference latency) and whether the architecture is feasible for the target deployment environment (cloud/edge/embedded).	*Compute requirements determine deployability; edge constraints are rarely reported yet are critical for real-world adoption.*	Methods—Implementation

## Data Availability

No new data were created or analyzed in this study. The review is based exclusively on previously published peer-reviewed literature. Data sharing is not applicable to this article.

## Abbreviations

The following abbreviations are used in this manuscript:

AE	Acoustic Emission
BNN	Bayesian Neural Network
CBM	Condition-Based Maintenance
CNN	Convolutional Neural Network
CP	Conformal Prediction
CRPS	Continuous Ranked Probability Score
CUSUM	Cumulative Sum Control Chart
DA	Domain Adaptation
DANN	Domain-Adversarial Neural Network
DAQ	Data Acquisition (System)
DL	Deep Learning
DS	Dempster–Shafer (Evidence Theory)
DT	Digital Twin
EKF	Extended Kalman Filter
EMG/sEMG	Electromyography/Surface EMG
FDD	Fault Detection and Diagnosis
FDI	Fault Detection and Isolation
FTC	Fault-Tolerant Control
GAN	Generative Adversarial Network
GNN	Graph Neural Network
GP	Gaussian Process
GRU	Gated Recurrent Unit
HIL	Hardware-in-the-Loop
HMM	Hidden Markov Model
IIoT	Industrial Internet of Things
IMM	Interacting Multiple Model
IMU	Inertial Measurement Unit
LLM	Large Language Model
LSTM	Long Short-Term Memory
MAE	Mean Absolute Error
MAPE	Mean Absolute Percentage Error
MC	Monte Carlo (Dropout)
MDS	Minimum Disclosure Set
ML	Machine Learning
MPIW	Mean Prediction Interval Width
MSCA	Motor Current Signature Analysis
NTP	Network Time Protocol
ODE	Ordinary Differential Equation
OOD	Out-of-Distribution
PF	Particle Filter
PHM	Prognostics and Health Management
PICP	Prediction Interval Coverage Probability
PINN	Physics-Informed Neural Network
PRISMA-ScR	Preferred Reporting Items for Systematic Reviews and Meta-Analyses—Scoping Reviews Extension
PTP	Precision Time Protocol (IEEE 1588)
ReLU	Rectified Linear Unit
RMSE	Root Mean Square Error
RTD	Resistance Temperature Detector
RUL	Remaining Useful Life
SCADA	Supervisory Control and Data Acquisition
SHM	Structural Health Monitoring
SNN	Spiking Neural Network
SOH	State of Health
SSM	State Space Model
TL	Transfer Learning
UKF	Unscented Kalman Filter
UQ	Uncertainty Quantification
VAE	Variational Autoencoder
WT	Wind Turbine
XAI	Explainable Artificial Intelligence
